# Three-dimensional evaluation and optimization of breast cancer rehabilitation science popularization videos on mainstream video platforms: a cross-sectional study in Mainland China

**DOI:** 10.3389/fdgth.2026.1970196

**Published:** 2026-10-14

**Authors:** Siqi Wu, Chang Chen, Ge Chen, Fuyou Wang, Jianxiong Wang

**Affiliations:** 1 Rehabilitation Medicine Department, The Affiliated Hospital of Southwest Medical University, Luzhou City, Sichuan Province, China; 2 Department of Traditional Chinese Medicine Rehabilitation, Jiangbei Campus, The First Affiliated Hospital of Army Medical University (Third Military Medical University), Chongqing, China; 3 Department of Orthopaedics, The Affiliated Hospital of Southwest Medical University, Luzhou City, Sichuan Province, China; 4 Department of Traditional Chinese Medicine Rehabilitation, Jiangbei Branch of The First Hospital Affiliated to Army Medical University (Third Military Medical University), Chongqing, China; 5 Department of Rehabilitation Medicine, Sichuan Clinical Research Center for Cancer, Sichuan Cancer Hospital & Institute, Sichuan Cancer Center, University of Electronic Science and Technology of China, Chengdu, China

**Keywords:** breast neoplasms, cancer rehabilitation, health literacy, lymphedema, social media, TikTok

## Abstract

**Background:**

With the growing number of breast cancer survivors, rehabilitation has become a critical component of comprehensive cancer care. Mainstream video platforms have emerged as a primary channel for patients to access rehabilitation information, yet the quality of such content remains unevaluated. This study aimed to systematically assess the quality and dissemination characteristics of breast cancer rehabilitation-related videos on TikTok, MicroBlog, and Today’s Headline.

**Method:**

A cross-sectional design was employed. From June to July 2026, videos related to breast cancer rehabilitation were retrieved on three platforms using an account-free search mode. 450 valid samples (150 per platform) were selected and a three-dimensional evaluation system was established: content scientificity (modified DISCERN scale), dissemination presentation (Global Quality Scale), and source credibility (JAMA scale). Overall quality was the arithmetic mean of the three dimensions. Six evaluators conducted scoring; inter-rater reliability was tested by ICC(2,k). *Post hoc* power analysis confirmed sufficient power (>0.99) for detecting medium-effect differences.

**Result:**

The overall quality score was 2.74 ± 0.85, with a pass rate (≥3.5) of only 19.8%; a sensitivity analysis using alternative cutoffs (≥3.0 and ≥4.0) yielded pass rates of 42.4% and 6.7%, respectively, confirming the robustness of the low-quality conclusion. TikTok's overall quality, content scientificity, and source credibility scores were significantly higher than MicroBlog and Today's Headlines (all *P* < 0.05, *η*^2^ = 0.11–0.16), a difference partly attributable to TikTok's higher proportion of certified physicians. One-way ANOVA revealed significant overall differences among four creator types (F = 128.6, *P* < 0.001, *η*^2^ = 0.46), with Tukey *post hoc* tests confirming certified physicians > rehabilitation nurses > health institutions > ordinary users. Subgroup analysis showed that sexual/reproductive rehabilitation videos had the lowest quality (2.45 ± 0.91). A weak negative association was observed between the engagement composite and quality (r = −0.18, 95% CI: −0.27 to −0.09, *P* < 0.001).

**Conclusion:**

The overall quality of breast cancer rehabilitation popular science videos on Chinese mainstream platforms was low. Patients should prioritize content from certified physicians and health institutions that cite guidelines and present balanced information. Platform-specific regulation and greater participation by certified oncologists and rehabilitation specialists are urgently needed.

**Level of evidence:**

Level II; Cross-Sectional Study

## 1 Background

Video-sharing platforms have become a dominant source of health information for the general public, with short videos combining visual demonstration, narration, and music to enhance engagement and retention (1). However, the quality of health-related videos on these platforms is highly variable, and content is often driven by entertainment and commercial interests rather than educational rigor (2, 3).

Breast cancer is the most commonly diagnosed malignancy among women in China, with approximately 420,000 new cases annually (4). The five-year survival rate has exceeded 83%, creating a rapidly growing population of long-term survivors (5). For these survivors, rehabilitation is an integral component of comprehensive care, encompassing upper-limb functional recovery, lymphedema management, psychosocial support, nutritional and exercise guidance, sexual and reproductive health, and body image restoration (6). Published guidelines emphasize that early, structured rehabilitation is crucial (7). Nevertheless, patient awareness remains inadequate: common misconceptions—such as “avoid all arm movement after surgery,” “lymphedema is irreversible,” and “pregnancy after breast cancer is unsafe"—are pervasive and can lead to avoidable disability and reduced quality of life (8). Timely, evidence-based rehabilitation education can significantly improve functional outcomes (9).

TikTok (Douyin), MicroBlog (Weibo), and Today's Headline (Toutiao) are the three dominant video platforms in China, each with over 500 million users and distinct content governance ecosystems. TikTok maintains a rigorous medical account certification system and content audit team, whereas MicroBlog and Today's Headline rely on relatively lenient interest-based authentication (10, 11). These regulatory differences directly shape the reliability of health information accessible to the public. Data from the Cyberspace Administration of China indicates that video platforms remove approximately 714 items of illegal medical content daily, 62.3% involving exaggerated therapeutic claims and false advertising—an issue particularly acute in oncology (12).

While prior studies have evaluated video quality for liver cancer (13), cervical cancer (14), and chemotherapy (15), no study has systematically assessed breast cancer rehabilitation content across multiple Chinese video platforms. Given the large survivor population, multidimensional rehabilitation needs, and prevalence of misinformation, this gap is clinically significant. The purpose of this study was to evaluate the quality, dissemination characteristics, and influencing factors of breast cancer rehabilitation videos on TikTok, MicroBlog, and Today's Headline in mainland China.

## 2 Methods

A cross-sectional study was conducted from June to July 2026. An account-free search mode was used throughout to avoid algorithm recommendation bias.

### 2.1 Platform selection

TikTok (http://www.douyin.com), MicroBlog (http://www.weibo.com), and Today's Headline (http://www.toutiao.com) were selected as the three most popular video platforms in Chinese mainland. YouTube was not included because it is not accessible to the general public in mainland China without specialized software, and therefore it is not a mainstream channel through which Chinese breast cancer survivors obtain health information. The three selected platforms have divergent regulatory mechanisms that provide meaningful comparative value, and all support short videos, graphic-text videos, and informational video formats.

### 2.2 Keyword determination

The following Chinese keywords were used: “乳腺癌康复” (breast cancer rehabilitation), “乳腺癌术后锻炼” (postoperative breast cancer exercise), “淋巴水肿” (lymphedema), “乳腺癌术后恢复” (postoperative recovery), “乳腺癌心理康复” (psychological rehabilitation), “乳腺癌营养” (nutrition for breast cancer patients), and “乳腺癌性生活” (sexual activity in breast cancer patients). Synonymous terms were supplemented through each platform's search association function.

Inclusion criteria: (1) content focuses on core breast cancer rehabilitation domains; (2) duration ≥ 10 s; (3) plays normally without significant audio/visual corruption; (4) not pure advertising.

Exclusion criteria: (1) duplicate content (video-text overlap ≥ 80%); (2) pure entertainment or off-topic content; (3) creator information unidentifiable; (4) illegal online diagnosis/treatment or unauthorized patient diversion.

### 2.3 Sample size, evaluation system, and video evaluation process

Referring to similar multi-platform health video evaluation studies, 150 valid samples per platform (450 total) were selected using the “top 300 by relevance + random sampling” method. Detailed random-sampling procedure: after retrieving search results sorted by platform default relevance, the top 300 entries for each platform were exported; each entry was assigned a unique random integer generated via the Excel RANDBETWEEN function. Entries were sorted by this random number, and the first 150 videos satisfying inclusion criteria were retained. A PRISMA-style flow diagram (Figure 1) documents screening from initial 1,428 retrieved records down to the final 450 analysed videos.

**Figure 1:**
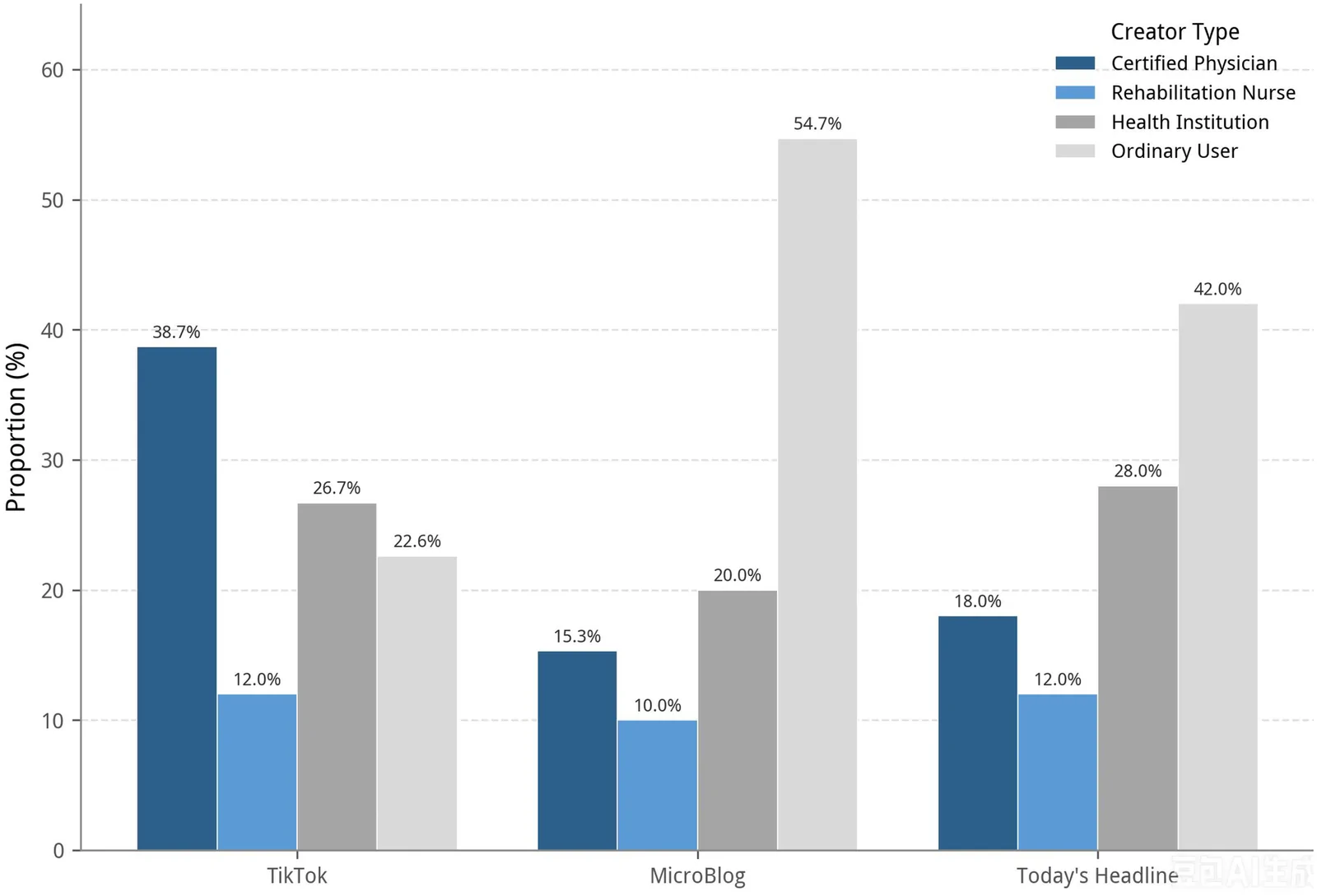
Flow diagram of video screening and selection. Records identified through platform searches (*n* = 1,428) were screened against inclusion/exclusion criteria; the top 300 eligible videos per platform (*n* = 900) formed the sampling frame, from which 150 videos per platform (*n* = 450) were selected by computer-generated random numbers.

A *post hoc* power analysis using G*Power 3.1 confirmed that, with a medium effect size (f = 0.25), *α* = 0.05, and 450 total samples, the statistical power exceeded 0.99 for both three-group (platform) and four-group (creator type) one-way ANOVA comparisons, well above the conventional 0.80 threshold.

A three-dimensional evaluation system was constructed (Table 1). The modified DISCERN (mDISCERN) scale was adapted from the validated full-length 16-item DISCERN instrument, which was originally designed for written patient health information. For the specific context of short-form breast-cancer-rehabilitation popular-science videos, we retained 8 core items assessing: (1) clarity of aims, (2) achievement of aims, (3) accuracy and evidence-base, (4) completeness of key rehabilitation information, (5) balance of benefits and limitations, (6) acknowledgment of uncertainty, (7) clarity and understandability, and (8) overall quality. We removed 8 items related to treatment-option comparison and shared decision-making (original items 3–8 and 13–15), which are poorly suited for brief public-facing short-video content. This shortened mDISCERN adaptation is study-specific and lacks independent external validation in separate short-video datasets; only inter-rater reliability from our calibration phase is reported herein. The mDISCERN score is the arithmetic mean of the 8 retained items (1–5 Likert scale). The GQS evaluated audiovisual quality, comprehensibility, and format adaptability. The JAMA scale assessed creator qualifications and information traceability.

**Table 1:** Three-Dimensional evaluation system for breast cancer rehabilitation videos.

First-level dimension	Second-level dimension	Evaluation tool	Scoring criteria (1–5 points)
Content scientificity	Accuracy, completeness, and balance of rehabilitation information	mDISCERN Scale	5: Fully complies with breast cancer rehabilitation guidelines; complete coverage of core rehabilitation domains; objectively presents benefits/limitations of interventions; clearly notes contraindications and individual differences. 4: Basic guideline compliance; core information complete; good balance; mentions some precautions. 3: No serious medical errors; core information relatively complete; average balance; no explicit contraindication warnings. 2: Minor medical deviation; incomplete core information; poor balance. 1: Serious medical errors; missing core information; misleading claims (e.g., “cures lymphedema,” exaggerated efficacy).
Dissemination presentation	Audiovisual quality, comprehensibility, format adaptability	GQS Scale	5: Clear audio/video, smooth playback; professional terms explained accurately and accessibly; standardized text annotations suitable for platform format; exercise demonstrations with correct form. 4: Good audiovisual quality; terminology relatively clear; text annotations generally accurate. 3: Average audiovisual quality, no lag; terminology explained but insufficiently clear; no obvious text errors. 2: Poor audiovisual quality, occasional stuttering; terminology unexplained or vague; minor text deviation. 1: Unwatchable audiovisual quality; terminology misleading; serious text errors.
Source credibility	Creator qualifications and information traceability	JAMA Scale	5: Creator qualifications complete and traceable (platform medical certification); information sources clearly cited (guidelines, peer-reviewed literature). 4: Creator qualifications verifiable; information sources not sufficiently detailed. 3: Creator qualification labeling incomplete; sources mentioned but unverifiable. 2: Creator qualifications not indicated; sources not clearly mentioned. 1: False qualification labeling; unfounded or fabricated sources.

The mDISCERN scale was adapted from the original 16-item DISCERN instrument, retaining 8 core items and removing 8 items related to treatment-option comparison and shared decision-making (original items 3–8 and 13–15). Each dimension is scored on a 1–5 Likert scale. Overall quality = arithmetic mean of the three dimensions (equal weights, each 1/3).

A Likert 5-point rating system was used (1 = extremely poor, 5 = excellent). A score ≥ 3.5 was defined as qualified. On a 5-point scale, 3 represents “average/fair” and 4 represents “good”; the 3.5 threshold denotes “above-average” quality, consistent with prior health-video quality studies (13, 16). Sensitivity analysis using alternative cut-offs (≥3.0 and ≥4.0) was performed to test robustness of pass-rate estimates (Table 2). Overall quality was the arithmetic mean of the three dimensions with equal weights (each 1/3). The rationale for equal weighting: (1) mDISCERN, GQS, and JAMA capture conceptually distinct facets of popular-science video performance; (2) no established psychometric framework exists to assign differential weights for short-video health-content assessment; (3) equal weighting maximizes transparency and reproducibility for cross-study comparison.

**Table 2:** Sensitivity analysis of qualification rates under alternative quality cutoffs.

Cutoff	Qualified n	Qualification rate	TikTok	MicroBlog	Today's headline	Inter-platform gradient retained?
≥ 3.0	191	42.4%	56.0%	34.7%	36.7%	Yes (*P* < 0.001)
≥ 3.5 (primary)	89	19.8%	30.7%	15.3%	14.0%	Yes (*P* < 0.001)
≥ 4.0	30	6.7%	11.3%	4.0%	4.7%	Yes (*P* = 0.006)

Qualification = overall quality score (equal-weight mean of mDISCERN, GQS, and JAMA) at or above the stated cutoff. The direction and significance of platform and creator-type differences were unchanged across all three cutoffs, indicating robustness to cutoff selection.

A team of six evaluators (three breast oncologists, two rehabilitation physicians, one health communication researcher) conducted the evaluation. The process was as follows: (1) all evaluators independently watched each video in its entirety before scoring; (2) evaluators were blinded to each other's scores; (3) a standardized scoring sheet based on the three-dimensional system was used; (4) a 30-video pre-evaluation calibration phase was conducted, with discrepancies >1 point discussed collectively to reach consensus; (5) during formal evaluation, score discrepancies >1.5 points were resolved by group discussion; (6) the final score for each dimension was the mean of all six evaluators’ scores; and (7) all evaluations were completed within a four-week period (June 15–July 13, 2026) to minimize evaluator drift. Inter-rater reliability ICC(2,k) = 0.84 (95% CI: 0.80–0.88) was calculated only on the 30-video calibration sample; this ICC represents agreement during rater training, and independent single-rater raw scores for the full main-study sample were not archived to compute a second full-sample ICC.

### 2.4 Data collection

Data collected included: (1) basic video information (upload time, duration, creator type, certification status); (2) interaction indicators (views, likes, comments, shares); (3) three-dimensional evaluation scores; (4) core rehabilitation domain. Data were aggregated in Microsoft Excel (Microsoft 2026, USA) and double-verified by two researchers.

### 2.5 Statistical analysis

Statistical analysis was performed using SPSS 26.0 (IBM, Armonk, NY). Descriptive statistics summarized sample characteristics. Because the social-media engagement variables (views, likes, comments, shares) were strongly right-skewed (Shapiro–Wilk *P* < 0.001), both medians with interquartile ranges and means with standard deviations are reported, and log10-transformed values were used in parametric analyses; the very high inter-correlations among views, likes, and shares (r > 0.99) were verified against raw platform metadata by two independent researchers before being combined into the z-score engagement composite. The Shapiro–Wilk test assessed normality of quality scores. One-way ANOVA compared quality scores across the three platforms and across the four creator types, with F-statistics, *η*^2^ effect sizes, and 95% confidence intervals reported. Tukey's HSD *post hoc* test was used for pairwise comparisons, with adjusted *P*-values and Cohen's d effect sizes. Inter-rater reliability was assessed using ICC(2,k) (two-way random effects, absolute agreement) on the calibration subset. Pearson correlation analysis (on log-transformed engagement variables) explored associations with quality, with r-values and 95% CIs. Multiple linear regression identified factors associated with video quality (*α* = 0.05); given the cross-sectional design, results are interpreted as associations rather than causal effects. The model included six independent variables selected *a priori* based on clinical relevance and bivariate findings: (1) certified physician creator status (binary), (2) video duration (continuous, per 10 s), (3) TikTok platform (binary), (4) engagement composite (continuous), (5) time elapsed since upload (continuous, months), included because older videos accumulate more views, and (6) content category (rehabilitation-focused vs. treatment-focused). Variance inflation factors (VIF) were examined for multicollinearity. Sensitivity analyses used alternative quality cutoffs (≥3.0 and ≥4.0) and an alternative weighting scheme. A subgroup one-way ANOVA compared overall quality across the six rehabilitation content domains.

## 3 Results

A total of 1,428 results were retrieved (TikTok: 478, MicroBlog: 506, Today's Headline: 444). After screening, 450 valid samples were included (150 per platform).

### 3.1 General features

On TikTok, certified oncologists/breast surgeons were the largest creator group (*n* = 58, 38.7%), followed by health institutions (*n* = 40, 26.7%), rehabilitation nurses (*n* = 18, 12.0%), and ordinary users (*n* = 34, 22.6%) (Figure 2). On MicroBlog, ordinary users dominated (*n* = 82, 54.7%), followed by health institutions (*n* = 30, 20.0%), certified physicians (*n* = 23, 15.3%), and rehabilitation nurses (*n* = 15, 10.0%). On Today's Headline, ordinary users were most prevalent (*n* = 63, 42.0%), followed by health institutions (*n* = 42, 28.0%), certified physicians (*n* = 27, 18.0%), and rehabilitation nurses (*n* = 18, 12.0%).

**Figure 2:**
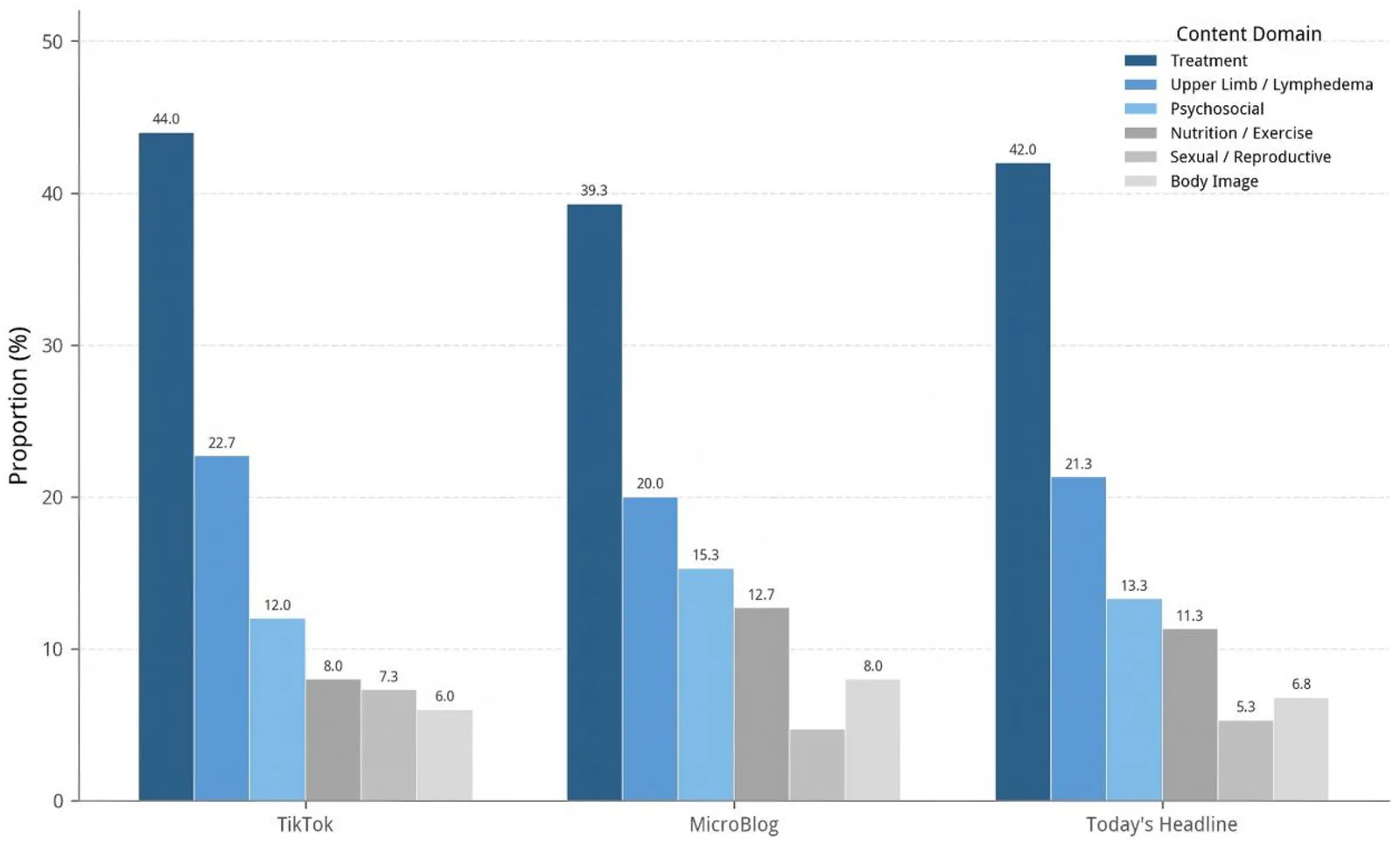
Proportion of creator types across three platforms (%).

Comparative analysis is shown in Table 3. All engagement variables were strongly right-skewed (Shapiro–Wilk *P* < 0.001); the overall medians (IQR) were 12,800 (5,200–28,600) views, 485 (186–1,024) likes, 42 (18–86) comments, and 36 (14–78) shares. TikTok had significantly higher views, likes, and shares than MicroBlog and Today's Headlines (all *P* < 0.05). TikTok's video duration (55.8 ± 24.6 s) was significantly shorter than MicroBlog (70.2 ± 28.9 s) and Today's Headline (65.7 ± 30.5 s) (both *P* < 0.05).

**Table 3:** General statistics by platform (x¯ ± s).

Platform	Upload time (months)	Duration (s)	Views	Likes	Comments	Shares
TikTok (*n* = 150)	6.2 ± 2.1	55.8 ± 24.6	16,842 ± 5,230	728 ± 268	52 ± 23	48 ± 19
MicroBlog (*n* = 150)	8.5 ± 3.1	70.2 ± 28.9	11,256 ± 4,120	384 ± 175	68 ± 27	32 ± 14
Today's headline (*n* = 150)	7.8 ± 2.8	65.7 ± 30.5	12,890 ± 4,450	462 ± 203	41 ± 19	36 ± 16
All (*n* = 450)	7.5 ± 2.7	63.9 ± 28.3	13,663 ± 4,620	525 ± 222	54 ± 23	39 ± 17
F-statistic	32.1	12.8	45.6	52.3	2.1	18.4
*η* ^2^	0.13	0.05	0.17	0.19	0.01	0.08

One-way ANOVA was used for overall comparison across three platforms. η^2^ = eta-squared (effect size). TikTok had significantly higher views, likes, and shares than MicroBlog and Today's Headline (all Tukey-adjusted *P* < 0.05). TikTok video duration was significantly shorter than the other two platforms (both *P* < 0.05). Comments showed no significant inter-platform difference (*P* = 0.125).

### 3.2 Quality assessment

The overall quality score across all platforms was 2.74 ± 0.85, with a qualified rate of 19.8%. One-way ANOVA revealed significant differences among platforms in overall quality (F = 28.4, *P* < 0.001, *η*^2^ = 0.11), mDISCERN (F = 35.2, *P* < 0.001, *η*^2^ = 0.14), and JAMA (F = 42.1, *P* < 0.001, *η*^2^ = 0.16), but not GQS (F = 2.1, *P* = 0.125, *η*^2^ = 0.01). Tukey *post hoc* tests confirmed TikTok scored significantly higher than MicroBlog and Today's Headline in overall quality, mDISCERN, and JAMA (all adjusted *P* < 0.05, Cohen's d = 0.62–0.85) (Table 4).

**Table 4:** Quality scores by platform (x¯ ± s).

Platform	Overall quality	mDISCERN	GQS	JAMA	Pass rate (%)
TikTok (*n* = 150)	3.15 ± 0.78 *	3.08 ± 0.72 *	3.12 ± 0.81	3.25 ± 0.76 *	30.7 *
MicroBlog (*n* = 150)	2.48 ± 0.81	2.31 ± 0.79	2.85 ± 0.84	2.28 ± 0.82	15.3
Today's headline (*n* = 150)	2.62 ± 0.83	2.47 ± 0.80	2.94 ± 0.82	2.45 ± 0.79	14.0
All (*n* = 450)	2.74 ± 0.85	2.62 ± 0.83	2.97 ± 0.83	2.66 ± 0.84	19.8
F-statistic	28.4	35.2	2.1	42.1	—
η^2^	0.11	0.14	0.01	0.16	—

One-way ANOVA was used for overall comparison across three platforms; Tukey's HSD *post hoc* test was used for pairwise comparisons. η^2^ = eta-squared (effect size). *P < 0.05 vs. MicroBlog and Today's Headline (Tukey-adjusted).

Among content themes, treatment-related videos were most common (TikTok: 44.0%, MicroBlog: 39.3%, Today's Headline: 42.0%), followed by upper-limb function/lymphedema (20.0%–22.7%). Sexual and reproductive rehabilitation content was scarcest (4.7%–7.3%) (Figure 3).

**Figure 3:**
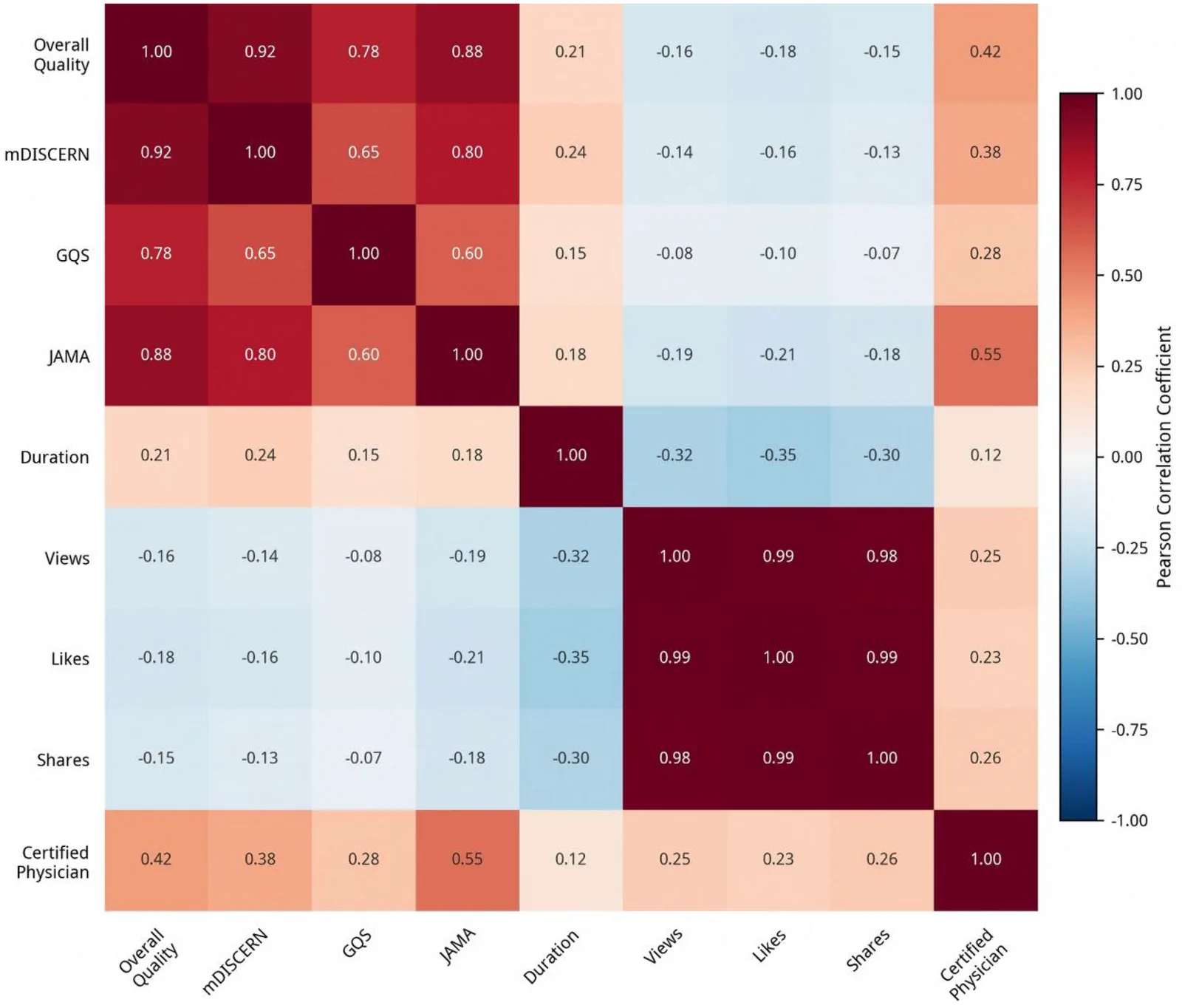
Proportion of rehabilitation content domains across three platforms (%).

One-way ANOVA revealed significant overall differences among the four creator types in overall quality (F = 128.6, *P* < 0.001, *η*^2^ = 0.46), mDISCERN (F = 115.3, *P* < 0.001, *η*^2^ = 0.44), and JAMA (F = 142.8, *P* < 0.001, *η*^2^ = 0.49). Tukey *post hoc* tests confirmed a consistent gradient: certified physicians > rehabilitation nurses > health institutions > ordinary users (all adjusted *P* < 0.05, Cohen's d = 0.58–2.15) (Table 5).

**Table 5:** Quality scores by creator type (x¯ ± s).

Creator type	n	Overall quality	mDISCERN	GQS	JAMA
Certified physician	108	3.68 ± 0.62	3.72 ± 0.58	3.55 ± 0.61	3.78 ± 0.55
Rehabilitation nurse	51	3.24 ± 0.68	3.18 ± 0.65	3.42 ± 0.63	3.12 ± 0.70
Health institution	112	2.86 ± 0.71	2.75 ± 0.69	3.08 ± 0.67	2.75 ± 0.73
Ordinary user	179	2.05 ± 0.74	1.92 ± 0.71	2.48 ± 0.78	1.75 ± 0.69
F-statistic	—	128.6	115.3	52.4	142.8
η^2^	—	0.46	0.44	0.26	0.49

One-way ANOVA was used for overall comparison across four creator types; Tukey's HSD *post hoc* test was used for all pairwise comparisons. All pairwise differences were statistically significant (adjusted *P* < 0.05), with Cohen's d ranging from 0.58 to 2.15. η^2^ = eta-squared (effect size). The quality gradient was: Certified Physician > Rehabilitation Nurse > Health Institution > Ordinary User.

A pre-specified sensitivity analysis examined whether the qualification rate depended on the 3.5 cutoff. Using ≥3.0 as the threshold, 191 videos (42.4%) qualified; using ≥3.5, 89 videos (19.8%) qualified; and using ≥4.0, only 30 videos (6.7%) qualified. The inter-platform and inter-creator-type quality gradients remained directionally identical under all three cutoffs, indicating that the study's conclusions are robust to the choice of threshold (Table 2).

A subgroup analysis across six content domains (Table 6) revealed significant differences in overall quality (F = 3.42, *P* = 0.005, *η*^2^ = 0.04): upper-limb/lymphedema videos scored highest (2.86 ± 0.82), followed by treatment (2.78 ± 0.86), psychosocial (2.71 ± 0.79), nutrition/exercise (2.68 ± 0.84), and body-image content (2.52 ± 0.88), while sexual/reproductive rehabilitation videos scored lowest (2.45 ± 0.91). Tukey *post hoc* tests showed that sexual/reproductive videos scored significantly lower than upper-limb/lymphedema videos (adjusted *P* = 0.008, Cohen's d = 0.47).

**Table 6:** Overall quality scores by rehabilitation content domain (x¯ ± s).

Content domain	n	Overall quality	mDISCERN	JAMA	Qualified rate (≥3.5)
Upper limb/lymphedema	96	2.86 ± 0.82	2.74 ± 0.80	2.78 ± 0.83	24.0%
Treatment	188	2.78 ± 0.86	2.66 ± 0.84	2.70 ± 0.86	20.7%
Psychosocial	61	2.71 ± 0.79	2.58 ± 0.76	2.62 ± 0.81	18.0%
Nutrition/exercise	48	2.68 ± 0.84	2.55 ± 0.82	2.58 ± 0.80	16.7%
Body image	31	2.52 ± 0.88	2.38 ± 0.86	2.42 ± 0.85	12.9%
Sexual/reproductive	26	2.45 ± 0.91	2.28 ± 0.89	2.35 ± 0.88	7.7%
ANOVA	—	F = 3.42, *P* = 0.005, η^2^ = 0.04	F = 3.18, *P* = 0.008	F = 2.94, *P* = 0.013	—

One-way ANOVA across six content domains with Tukey HSD *post hoc* tests. Sexual/reproductive videos scored significantly lower than upper-limb/lymphedema videos (adjusted *P* = 0.008, Cohen's d = 0.47). Small subgroup sizes (*n* = 26–31 for body-image and sexual/reproductive domains) warrant cautious interpretation.

### 3.3 Correlation and regression analysis

Views, likes, and shares were strongly positively correlated (r > 0.99); these high correlations were independently verified against raw platform metadata by two researchers. An engagement composite was constructed by z-score standardizing each log-transformed metric (mean = 0, SD = 1) and computing their arithmetic mean. Overall quality showed a weak negative association with the engagement composite (r = −0.18, 95% CI: −0.27 to −0.09, *P* < 0.001); given the small magnitude of this correlation, this finding should be interpreted cautiously as a weak tendency for more engaging videos to have marginally lower quality, rather than evidence of a deterministic “paradox” (Figure 4).

**Figure 4:**
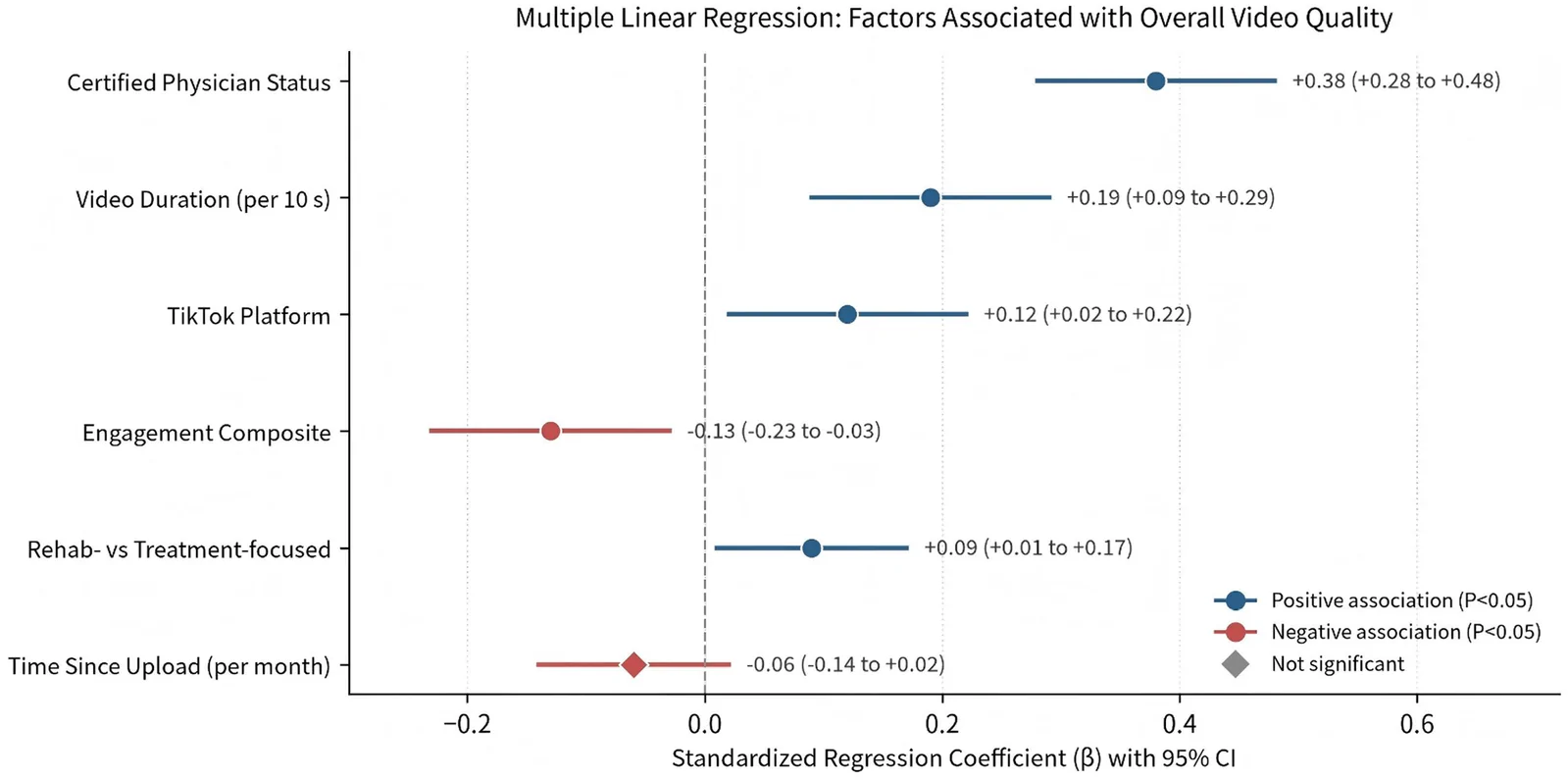
Correlation heatmap of video indicators across all platforms.

In the multiple linear regression model adjusted for upload time and content category (Table 7, Figure 5), certified physician status (standardized *β* = 0.38, 95% CI: 0.28–0.48, *P* < 0.001), longer video duration (*β* = 0.19, 95% CI: 0.09–0.29, *P* < 0.001), TikTok platform (*β* = 0.12, 95% CI: 0.02–0.22, *P* = 0.018), and rehabilitation-focused (vs. treatment-focused) content (*β* = 0.09, 95% CI: 0.01–0.17, *P* = 0.038) were positively associated with overall quality, whereas the engagement composite was weakly negatively associated (*β* = −0.13, 95% CI: −0.23 to −0.03, *P* = 0.012). Time since upload was not significantly associated with quality after adjustment (*β* = −0.06, *P* = 0.18). Of note, the TikTok coefficient attenuated from 0.19 to 0.12 after creator composition was included in the model, suggesting that the platform difference is partly mediated by TikTok's higher proportion of certified physicians. All VIF values were <2.5. The model explained 40.5% of the variance (R^2^ = 0.405, adjusted R^2^ = 0.397, F(6, 443) = 50.2, *P* < 0.001). As this is a cross-sectional study, these variables should be interpreted as factors associated with—not causal predictors of—video quality.

**Table 7:** Multiple linear regression analysis predicting overall video quality.

Predictor variable	B (95% CI)	Std. *β*	t	*P*-value
(Constant)	1.28 (1.02–1.54)	—	9.7	< 0.001
Certified physician status	0.78 (0.58–0.98)	0.38	7.6	< 0.001
Video duration (per 10s)	0.06 (0.03–0.09)	0.19	3.9	< 0.001
TikTok platform	0.24 (0.04–0.44)	0.12	2.4	0.018
Engagement composite (log, z-scored)	−0.10 (−0.18 to −0.02)	−0.13	−2.5	0.012
Time since upload (per month)	−0.02 (−0.05 to 0.01)	−0.06	−1.3	0.180
Rehabilitation- vs. treatment-focused	0.18 (0.01–0.35)	0.09	2.1	0.038

Multiple linear regression with overall quality score as the dependent variable (cross-sectional associations, not causal effects). B = unstandardized coefficient; CI = confidence interval; Std. β = standardized coefficient. Model fit: R^2^ = 0.405, adjusted R^2^ = 0.397, F(6, 443) = 50.2, *P* < 0.001. Certified Physician Status: 1 = certified physician, 0 = other. TikTok Platform: 1 = TikTok, 0 = MicroBlog or Today's Headline. Engagement Composite = z-scored mean of log10-transformed views, likes, and shares. All VIF values were < 2.5. The TikTok coefficient attenuated from β = 0.19 to β = 0.12 after adjustment for creator composition, suggesting partial mediation by certified-physician share.

**Figure 5:**
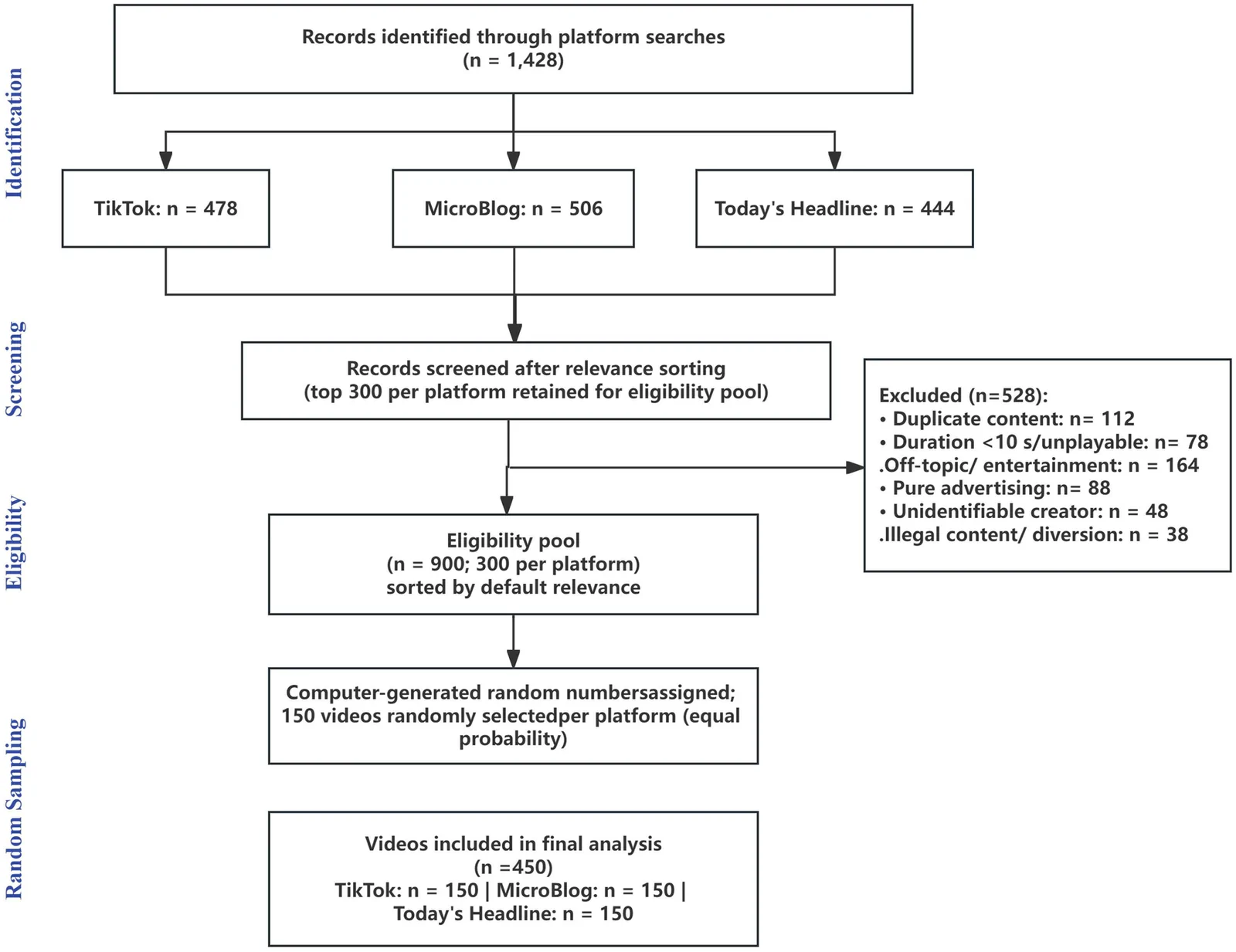
Forest plot of standardized regression coefficients (*β*) from multiple linear regression analysis predicting overall video quality. Positive *β* values indicate positive associations; negative *β* values indicate negative associations. Error bars represent 95% confidence intervals.

## 4 Discussion

This study is the first to systematically evaluate the quality of breast cancer rehabilitation videos across three major Chinese video platforms. Our findings reveal that the overall quality is alarmingly low (2.74 ± 0.85; pass rate 19.8%), a finding consistent with prior evaluations of liver cancer videos (21.3% pass rate) (13), uterine fibroid videos (22.5%) (17), and cancer rehabilitation content on YouTube (18). This is particularly concerning given that breast cancer survivors constitute one of the largest and most information-seeking patient populations, and rehabilitation decisions directly affect their functional recovery and quality of life.

Notably, some studies in the literature have reported comparatively higher quality scores. For example, Hu et al. found median LassVQ scores of 3.77 on YouTube and 3.66 on Douyin for general health educational short videos (19), and Wang et al. reported that 36%–40% of sleep-disorder videos on TikTok achieved GQS or modified DISCERN scores ≥4, with physician creators accounting for 64% of the sample (20). Several factors may explain why breast cancer rehabilitation videos in our study scored lower. First, the creator composition differed markedly: physician creators accounted for only 24.0% (108/450) of our overall sample, whereas the higher-scoring studies were dominated by medical professionals. Second, breast cancer rehabilitation is inherently multidimensional—spanning upper-limb function, lymphedema, psychosocial, nutritional, sexual/reproductive, and body-image domains—and the predominantly very short videos in our sample (median 55.8 s on TikTok) rarely allowed systematic coverage of these domains, whereas the higher-scoring studies addressed comparatively focused topics or used longer videos (median 88–189 s). Third, the emotional and commercial sensitivity surrounding cancer survivorship may attract more unqualified creators and disguised advertising. These contrasts suggest that disease complexity, video duration, and—most importantly—the proportion of professional creators may be important factors contributing to the observed quality differences; however, given the cross-sectional design, these interpretations remain associative.

The mDISCERN dimension scored lowest (2.62 ± 0.83), reflecting widespread deficiencies in the accuracy, completeness, and balance of rehabilitation information. Common issues included oversimplified exercise prescriptions without contraindication warnings, failure to distinguish between post-mastectomy and post-lumpectomy rehabilitation protocols, and omission of lymphedema risk-reduction strategies. The scarcity of sexual and reproductive rehabilitation content (4.7%–7.3%) is especially noteworthy: fertility preservation, sexual function after endocrine therapy, and safe contraception are among the most frequently asked questions by young breast cancer patients (21), yet they are severely underrepresented. This “utilitarian skew"—prioritizing treatment topics that attract clicks over nuanced rehabilitation and prevention domains—mirrors findings in uterine fibroid (17) and chemotherapy (22) video evaluations.

Content creator composition is strongly associated with video quality, and platform governance may act as a structural moderator by shaping the mix of creators. TikTok significantly outperformed MicroBlog and Today's Headline in overall quality, content scientificity, and source credibility (*η*^2^ = 0.11–0.16). This platform quality gradient (TikTok > other platforms) is consistent with prior cross-platform studies of liver cancer (13) and atrial septal defect (23) videos. Importantly, our adjusted regression showed that the TikTok coefficient attenuated substantially (from *β* = 0.19 to *β* = 0.12) once creator composition was controlled for, indicating that TikTok's apparent quality advantage is partly explained by its higher proportion of certified physicians (38.7% vs. 15.3% on MicroBlog), rather than platform effects alone. The remaining advantage is plausibly related to TikTok's stricter medical account certification and content audit mechanisms (24). TikTok's pass rate (30.7%) was nearly double that of MicroBlog (15.3%) and Today's Headline (14.0%); however, even on TikTok, nearly 70% of videos failed to meet the quality threshold, indicating that platform-level regulation alone is insufficient.

Consistent with prior studies demonstrating that certified physician-created videos achieve significantly higher quality than non-professional content (25, 26), certified physician-created videos in our study achieved significantly higher quality than other creator types (*η*^2^ = 0.46–0.49, Cohen's d = 0.58–2.15). This underscores the indispensable role of professional creators. Nevertheless, even certified physician videos sometimes lacked comprehensiveness—many focused narrowly on treatment selection while neglecting multidimensional rehabilitation guidance. Rehabilitation nurses, though underrepresented, demonstrated good content quality, highlighting an underutilized resource.

A weak negative association was observed between engagement and quality (r = −0.18, 95% CI: −0.27 to −0.09), consistent in direction with the weak quality-engagement associations reported in Takotsubo syndrome videos (27), CAR-T therapy content (28), and broader analyses of medical information ecosystems (29). Because the magnitude of this correlation is small, we refrain from describing it as a confirmed “paradox”; it indicates only a slight tendency for highly engaging videos to have marginally lower quality at the aggregate level, and it does not imply that individual high-engagement videos are necessarily low-quality. Several mechanisms could contribute to this weak tendency: (1) platform algorithms prioritize rapid engagement, which may favor emotionally charged content over nuanced evidence-based material (29); (2) the public may lack the expertise to distinguish quality and may interact more with simple, emotionally resonant content (16); and (3) high-quality content tends to be more complex. Breast-cancer-specific factors—the emotional intensity of a cancer diagnosis, the commercialization of survivorship products, and the personal nature of body-image and fertility concerns—may further amplify the appeal of anecdotal content.

In the current short-video era, algorithm-driven content recommendation and fragmented viewing habits profoundly shape the information environment for breast cancer survivors. The consequences for patients are multifaceted. In terms of health beliefs, repeated exposure to low-quality content may foster unrealistic expectations or unnecessary fear, distorting patients’ understanding of their prognosis. In terms of rehabilitation behaviors, incorrect exercise demonstrations or contraindicated advice may lead to delayed functional recovery or exacerbation of lymphedema. In terms of medical decision-making, patients may defer or reject standard evidence-based therapy in favor of unproven alternatives promoted through engaging but inaccurate videos (30, 31).

Healthcare professionals must proactively adapt to this evolving information environment (32, 33). First, structured training in health communication and short-video production should be integrated into continuing medical education. Second, professionals should leverage platform-native formats—such as step-by-step exercise demonstrations and subtitle annotation—to enhance accessibility without compromising rigor. Third, collaboration between professional societies and video platforms to build authoritative content pools can help counterbalance the visibility advantage of low-quality material. Fourth, healthcare professionals should actively engage in comment sections to correct misinformation in real time. Finally, rehabilitation nurses and patient navigators should be empowered as key content creators.

For patients and social media users, our findings offer practical guidance on which videos to trust. Patients should prioritize videos that: (1) are created by certified physicians, rehabilitation nurses, or recognized health institutions (verified by platform medical certification badges); (2) cite clinical guidelines or peer-reviewed literature as information sources; (3) present balanced information, including both benefits and limitations/uncertainties, rather than making absolute claims; (4) avoid sensational titles such as “cure,” “miracle,” or “one trick fixes”; (5) cover multidimensional rehabilitation topics rather than focusing solely on treatment; and (6) encourage consultation with healthcare professionals rather than replacing medical advice. Conversely, patients should be cautious of videos that make unsubstantiated therapeutic claims, promote unproven products, or are created by unverified accounts. When in doubt, patients should discuss online information with their oncologist or rehabilitation specialist.

Our findings have several practical implications for platforms and creators. Platforms should implement mandatory qualification certification for medical rehabilitation content creators and establish tiered review mechanisms. Professional oncology and rehabilitation societies should develop standardized, patient-friendly rehabilitation video libraries. Algorithms should be adjusted to prioritize traffic for certified, high-quality content.

This study has limitations. First, the sample was limited to 450 videos and a single disease entity. Second, only mainland Chinese platforms were included; YouTube was deliberately excluded because it is not accessible to the general public in mainland China, though cross-cultural comparisons with YouTube and Instagram would be valuable for future international studies. Third, the relevance-based sampling approach, even with account-free searching and random subsampling, cannot fully eliminate biases originating from platform proprietary ranking algorithms. Fourth, we performed exploratory subgroup analysis across rehabilitation content domains; these subgroup comparisons should be interpreted as descriptive rather than confirmatory. Fifth, the shortened mDISCERN scale is a study-specific adaptation of the original DISCERN tool. Although we preserved core items of the original instrument and achieved good inter-rater reliability within our calibration sample, this modified scale has not been subjected to independent external construct or criterion validation on separate medical-short-video datasets. Further psychometric evaluation is required before wider adoption of this mDISCERN version. Sixth, cross-sectional design restricts us to reporting associations; no causal conclusions can be drawn.

## 5 Conclusion

The overall quality of breast cancer rehabilitation popular science videos on mainstream Chinese video platforms is low, with insufficient scientific accuracy, incomplete rehabilitation domain coverage, and variable source credibility; this conclusion was robust to alternative quality cutoffs in sensitivity analyses. Certified physician-created videos were associated with higher quality, but professional creators often lack comprehensiveness. A weak negative association between engagement and quality suggests a slight tendency for highly engaging content to be of marginally lower quality, and the apparent platform advantage of TikTok was partly explained by its higher share of certified physicians. Patients should prioritize content from certified physicians and health institutions that cite guidelines and present balanced, non-sensational information. Strengthening platform-specific regulation, mandating creator qualification certification, empowering rehabilitation professionals with health communication training, and optimizing algorithmic prioritization may help improve the quality of breast cancer rehabilitation information and support the health of millions of survivors.

## Data Availability

The raw data supporting the conclusions of this article will be made available by the authors, without undue reservation.
